# Possible Interventions for Preventing the Development of Psychopathic Traits among Children and Adolescents?

**DOI:** 10.3390/ijerph19010409

**Published:** 2021-12-31

**Authors:** Gunnar Bjørnebekk, Dagfinn Mørkrid Thøgersen

**Affiliations:** 1 Department of Special Needs Education, University of Oslo, P.O. Box 1140, 0318 Oslo, Norway; 2 The Norwegian Center for Child Behavioral Development, Essendrops gt. 3, 0368 Oslo, Norway; d.m.thogersen@nubu.no

**Keywords:** callous-unemotional traits, personalised treatment, conduct disorder, comorbidity

## Abstract

Individuals with the combination of psychopathy and severe conduct disorder often get in a lot of trouble from their early childhood, and can cause great suffering and problems for other people and their immediate environment. Their antisocial behaviour has a tendency to develop into a chronic pattern early in life, and the treatment prognosis in adulthood is poor. A large proportion of serious violent crimes in society can be attributed to this group of perpetrators. Until recently, it has been unclear whether traits of this type can be prevented or changed, so that these individuals and their surroundings can benefit from targeted treatments at an early stage. To reduce serious crime in a society, it is very important to develop effective measures for this particular group. A lack of empathy, indifference to others, and a lack of concern about their own performance appear to be key early signs in children and adolescents with persistent behavioural problems and more serious norm violations who continue into a criminal career upon reaching adulthood. These characteristics have been termed callous−unemotional (CU) traits, and they are considered to be a precursor to psychopathic traits in adulthood. In recent years, several studies have evaluated the degree to which treatments that have been proved effective for children and adolescents with severe behavioural problems also show effectiveness for children and adolescents with CU traits. Interventions specifically tailored to children with CU traits have also been developed with the aim of directly changing the ongoing development of this precursor to psychopathy. In this paper, we will address the extent to which current evidence-based treatment methods developed for children and adolescents with behavioural difficulties are equally effective when a child has CU traits. We will also take a closer look at the effects of interventions designed to change this trait. There will be a discussion regarding what seems relevant for a change in the trait itself, as well as a change in their antisocial behaviour.

## 1. Introduction

Individuals with the combination of antisocial traits and severe conduct disorder (CD) are prone to get into a lot of trouble during their lives. They can also cause great suffering and problems for other people and their immediate environment. Surveys have shown that more than half of the most serious crimes in society and around 75% of violent crimes are committed by a small group of the population [1]; 15–20% of this criminal group and around 1% of a cohort can be characterised by how they, in addition to violating a number of norms, also show a lack of conscience and feelings for others and are indifferent to how they perform. For example, they may not care how well they do in a test at school. This subgroup is characterised by a more stable type of antisocial behaviour, with a higher rate of violent crime resulting in considerable harm to other people. Their actions also entail significant socioeconomic costs. Even though the group with severe CD and antisocial traits is relatively small, offering them treatment to prevent repetitive norm-breaking and destructive actions would be of great benefit to society.

Life-course research has shown that children who show early signs of both antisocial traits (impulsivity, narcissism, and low empathy) and antisocial behaviour tend to develop a chronic pattern of norm-transgressive behaviours [2,3]. Several studies indicate that, in adulthood, these individuals have a poorer treatment prognosis than if targeted measures had been implemented at an early age. To reduce serious crime in a society, it is important to develop effective measures for children and adolescents who show early signs of serious norm-transgressive behaviour. There is relatively widespread consensus that such signs can be identified early in life, but there are important moral arguments to avoid the diagnosis of antisocial personality disorder or the term psychopathy with respect to children and adolescents. Psychopathy is characterised by behavioural patterns and traits that, in most cultures and societies, are generally assessed to be undesirable [4]. However, some of these traits, such as impulsivity and egocentrism, are relatively normal in children and adolescents. Several important relational skills and ways of thinking are still subject to development in children and adolescents [5]. Several research studies have identified low empathy as a key early sign in children and adolescents with persistent behavioural problems, more serious norm violations, and continued criminal careers in adulthood [2]. The term callous−unemotional (CU) traits is used to designate these characteristics, and CU traits are considered to be a precursor to psychopathic traits in adulthood. Children and adolescents with CU traits are characterised by showing little concern for others, not caring how they perform on various tasks, and showing little remorse or guilt after norm violations. The characteristics of CU traits correspond to the interpersonal and emotional facets that denote antisocial personality disorder in adults. Other adult psychopathic facets, such as impulsivity and egocentrism, are not included in the CU designation, as they appear to be more associated with antisociality in general, than with CU. In the DSM-5 diagnostic system, CU traits are included as a subcategory of the conduct disorder diagnosis, and are therein termed limited prosocial emotions (LPE).

Historically, there have been periods when it has been considered a relative waste of time to treat or do anything about severe behavioural problems [6]. During the past decades, however, several evaluations have shown that there are effective treatment models for children and adolescents with these problems. Clinical trials have demonstrated that even though children and adolescents with severe behavioural problems are a challenging target group, several treatment models can provide significant reductions in problematic behaviour [7]. Interventions proven effective for children and adolescents with behavioural problems in general are now evaluated for their effectiveness when the child or adolescent in treatment also acts in ways that are characterised by low levels of remorse or guilt (i.e., behaviour related to CU). Furthermore, some research groups have developed interventions specifically aimed at changing the factors that increase the likelihood of developing psychopathic traits (e.g., “empathic-emotion recognition training”; [8]). Some of these interventions are offered as supplementary modules to established treatment, while others are distinct treatment programs by themselves.

In this paper, we will address the extent to which established evidence-based treatment methods developed for children and adolescents with behavioural problems are effective in the face of CU traits. We will discuss the treatment methods of Multisystemic therapy (MST) and Functional Family Therapy (FFT), as well as parent training programs such as parent management training—Oregon (PMTO). Furthermore, we will take a closer look at the effect of interventions designed to change the core components related to the development of psychopathic traits (such as CU). We will address what is linked to a reduction in either CU traits or antisocial behaviour, or both. To understand why and how various treatments can be effective for children with a combination of CU and severe behavioural problems, we will start with a brief description of CU traits and their development.

## 2. Definition and Development

### 2.1. “Callous Unemotional Traits”/Limited Prosocial Emotions

Life-course studies indicate that the severity and stability of antisocial behaviours relate to the age at which problematic behaviours become evident in the child. Children exhibiting antisocial behaviour in early childhood (3–10 years of age) are at increased risk of persistent problematic behaviour that spread from within the family to other areas (such as school, friends, and the neighbourhood). Moffitt [9,10] characterises this group as one with life-course persistent antisocial behaviour. For those where behaviour problems first appear in adolescence, antisocial behaviour tends to be limited to the teenage years, with a lower risk of the norm-breaking behaviour continuing into adulthood [9,11]. The Diagnostic and Statistical Manual of Mental Disorders (DSM) used this research to establish the “early starter” (before the age of 10) and “late starter” (older than 10) sub-groups for CD [12]. While diagnostically useful, there is considerable variability within both these groups with respect to the stability and severity of conduct problems [2,13]. Some young people whose behaviour does not become problematic until adolescence may end up in prison, where they establish ties with more serious criminals. Other late starters may become addicted to drugs and thereby develop more antisocial behaviour and personalities in adulthood. Research also indicates that some early starters cease their antisocial activities during adolescence and do not show similar problems later.

Early or emerging psychopathic traits might play an important role in explaining the variability within these subgroups. It appears that among the children who show early behavioural problems, those with clear signs of psychopathic traits are more likely to continue their antisocial behaviour into adulthood [14]. Fox et al. [15] have argued for including psychopathic traits in assessments of the prognoses for children whose behaviour is problematic from an early age. This is supported by studies showing that the group of children showing these traits also has a tendency to develop more serious and chronic problems that are characterised by serious norm transgressions and violent crime [16,17]. As noted earlier, the emotional aspect of psychopathy is most relevant as a predictor among children and youth with severe behaviour problems, and is included as specifier to the CD diagnosis, under the term Limited Prosocial Emotions (LPE) [12]. The specifier is used if at least two of the following four criteria have been present over the past 12 months in more than one relationship and in more than one context:

1. Lack of remorse or guilt
2. Lack of empathy
3. Unconcerned about performance in important areas
4. Shallow or deficient affect

Only 25–30% of children with severe CD meet the criteria for LPE, but the proportion may vary somewhat between different countries. A major British study of young people aged 5–16 years old, found 2% met the criteria for a severe CD and 46% of these also scored high for CU traits [18].

### 2.2. Central Characteristics of CU Traits

Children and adolescents who are diagnosed with CD and the CU specifier (LPE) are thought to differ from those who do not qualify for the specifier in a number of ways. According to the most comprehensive systematic literature review undertaken for CU traits, the CD + CU group show several emotional, cognitive, motivational, and social difficulties [2]. They exhibit more deviant values and social goals in social situations. Among other things, they think it is okay to use violence to achieve their goals and to blame others when they have done something wrong. They pay little attention to signs of fear, pain, and sadness in others, and in tests of moral transgressions they struggle to assess which actions might have negative consequences for others and which actions transgress the social rules in the relevant situation [2]. These limitations might help explain why children with behavioural problems and CU traits tend to develop a more serious and chronic pattern of antisocial behaviour compared to children with low-CU behavioural problems [19]. They are more likely to continue to be criminals in adulthood and to use violence that physically harms others to a greater extent. Furthermore, they appear to have a tendency to focus more on the positive aspects of using violence [20], use aggression on a more instrumental and planned basis [2], and have difficulty understanding or recognising the suffering of others [21].

### 2.3. CU as a Precursor of Psychopathy

If the treatment of CU is to reduce the likelihood of developing psychopathic traits, there must be an association between CU in children and psychopathic traits in adulthood. Furthermore, CU must be something that “does not pass by itself”, if prevention should be prioritized. The findings from longitudinal studies indicate that the interpersonal and affective traits are more stable than the antisocial character traits [22]. The latter appear to decline sharply with increasing age. The stability estimates of the affective and interpersonal traits vary relatively widely, however, and the time intervals examined range from a few months to 11 years. Lynam et al. [23] conclude that psychopathy appears to be just as stable as other personality traits. It is nonetheless important to point out that most children who score high for CU traits early in life do not develop psychopathy. Among the 20% who, at the age of 13, were assessed by their mothers to have the highest degree of psychopathic traits, only half of them fulfilled the criteria for a psychopathy diagnosis at the age of 24. However, only a very small proportion of those who received a low score at the age of 13 fulfilled the criteria for a psychopathy diagnosis at the age of 24. It is therefore easier to predict with a high degree of certainty who does not fulfil the diagnosis criteria, based on a low score for psychopathic traits at the age of 13. In brief, the research support that callous unemotional traits are relatively stable and that various measures of CU traits have the potential to identify a specific group of children and adolescents with more severe and persistent behavioural problems. These individuals’ cognitive and relational skills and abilities are still being developed, however, and before these are fully developed, adolescents may act in ways that resemble these traits. Research has found that scores on CU traits were by far the highest among 14-to-15 year-olds [24]. The risk of false positives should therefore be considered when assessing CU traits in adolescents, and so far, there is no easy way of screening for these traits to identify the children who will fulfil the criteria for psychopathy in adulthood.

### 2.4. Relationship between Parenting Practices and CU Traits

The core of CU traits appears to develop in the interactions with a child’s surroundings at an early age. Factors such as capacity for “empathic concern” [25], helping others [26], lying about naughty behaviours [27], and the ability to distinguish between right and wrong [28], are all developed at around the age of 2–3. The most important environmental aspect for children in this age group is the child’s parents and their interaction with the child [29]. Parental practices have therefore been viewed as an important factor in the development of CU traits.

Several international studies have investigated the relationship between parenting practices and CU traits in children, and so far, the research findings are ambiguous. A few studies have pointed to how a lack of parental affection can lead to CU traits in children [30,31]. The same applies to inconsistent upbringing or excessively strict parenting practices [32]. Other studies have shown the reverse, namely that CU traits in children contribute to excessively strict parenting practices [33]. In order to make sense of such contradictory findings, it can be helpful to view the relationship between CU traits and parenting practices from a transactional perspective, where CU traits and parenting can mutually influence each other over time. The interaction between a child and his/her parent(s) is of crucial importance to a child’s development in general, and to behavioural problems and CU traits in particular.

According to Patterson’s model of social interaction and learning (SIL), negative and escalating interaction patterns consisting of mutual coercion and tension between children and parents are the single most important factor in the development of behavioural difficulties [29]. These coercive interaction patterns occur in the family when the child’s aggressive behaviour is negatively reinforced. An example of this may be a child screaming and punching to get their way. In some families, coercive interactions between parents and the child are automated and become a central aspect of the family’s interchanges. In this way, Patterson’s SIL model takes into account that the child’s behaviour and the parents’ conduct mutually affect each other over time. Children in these families have a double handicap: they learn to use negative behaviour, threats, and force to get their way. At the same time, their development of social competence is weakened because they often get rejected by prosocial peers and teachers [34]. It is still uncertain, however, whether the coercive interaction pattern drives the development of antisocial behaviour in those who score high for CU. Coercive family processes seem to be more influential on the development of behavioural problems in those who do not have CU traits [35]. A systematic review assessed that the high-CU group seems far less affected by both strict upbringing practices and coercive interactions than the low-CU group [2]. They conclude that genetics, rather than hostile parenting practices, are likely to be the primary source of antisociality in the high-CU group. On the other hand, CU traits are likely to adversely affect parenting practices by promoting excessively strict parenting practices [33,36]. This may explain the findings of two studies in which strict parenting practices were linked to the child’s degree of CU traits later in life [37,38].

Even though research may indicate that CU traits in the child are more likely to adversely affect the parents’ competences than vice versa, it is still important to promote adequate parental skills and positive parenting practices towards children who score high for CU. Reducing excessively strict parenting practices and increasing parents’ positive involvement and affection might still be effective at reducing the development of antisocial behaviour in children and adolescents with CU traits. This notion is also supported by research findings showing that the “warmth” of the parents’ affection has a stronger impact on children with high CU traits. Among the children with high CU traits, studies have shown that observed parental warmth is negatively related to conduct problems [35], and that an affectionate upbringing reduces the degree of their behavioural problems over time [39]. Positive parenting skills (positive involvement, warm affection, and use of praise/reward) have also predicted the level and change in CU in several longitudinal studies [32,33,40]. Interventions that aim to increase parents’ positive involvement, affection, and the use of praise/reward can thus be assumed to be important for changing CU traits and antisocial behaviour.

O’Connor et al. [41] conducted an observational study in which they found that adolescents with a high degree of CU reacted to a greater extent with anger and irritation in interaction tasks with their parents when discussing conflictual topics compared to adolescents with a low degree of CU. The parents, on the other hand, were not found to change the degree of warmth/support or anger/irritation in relation to the young people’s degree of CU traits. It also seems that adolescents’ experience of conflict in the family, either between the parents or between a parent and the young person themselves, is an important risk factor for increased aggression among adolescents who score high for CU traits [42]. These findings show that the quality (“warmth”) of the parent−youth relationship and family conflicts matter to these young people, and indicate that improvements in these aspects of family relationships could positively affect their development.

## 3. Possible Early Preventive and Treatment Interventions

There are a number of well-established models for parent training and family treatment, and several of these have been proved effective for children and adolescents with behavioural problems [7,43]. In general, these programs are aimed at improving the child’s context as a developmentally supportive environment. For younger children, this takes place by teaching the parents new parenting strategies such as positive involvement, praise, and encouragement, as well as effective and non-conflictual setting of boundaries. In the treatment of adolescents, weight is still given to improving parenting practices, but the involvement of the young person in the treatment process is emphasised, and additional focus is put on important areas outside the family, such as relations with friends, school, and leisure activities. Detailed descriptions of the various effective programs are presented by various public clearing houses, such as the Blueprints website (https://www.blueprintsprograms.org/program-search/ (accessed on 6 November 2021)). It is important to ascertain whether these family-oriented treatments are appropriate to disrupt the development of CU and/or reduce the antisocial behaviour of children and youth with CU traits. Frick et al. [2] conclude that children who score high for CU traits have a poorer treatment prognosis. Out of the 20 studies reviewed, 18—i.e., almost 90%—reported that persons with a high level of CU traits showed poorer treatment outcomes than those who only had behavioural problems. Many of these studies were conducted in residential or correctional settings and some lacked a clear description of the treatment provided. A review focusing on well-established and promising treatment models is therefore warranted, and we will begin with a closer look at various promising preventive and treatment interventions for pre-adolescent children.

Parent training programs aimed at children with behavioural difficulties are intended to help parents meet the child with positive involvement, encouragement, and praise as the main means of ensuring improved cooperation with the child on day-to-day activities and chores. In these programs, parents also learn how to set boundaries for and respond to negative behaviour through age-appropriate and conflict-reducing strategies. As children with CU traits might be less influenced by negative consequences and more “indifferent” to whether they perform tasks in the right way, it is of interest to examine if the various elements of parent training have differential effects for these children. Hawes and Dadds [44] showed that children with high CU traits achieved just as much positive change as children with low CU traits when their parents learned ways to encourage prosocial behaviour. This was not the case when parents learned to discourage deviant behaviour through appropriate and contingent use of mild sanctions. Here, children with stable high CU traits showed a far less reduction of their problematic behaviour than children with low CU traits [45].

Families in which a child shows CU traits may need additional treatment components than those included in the regular parent training programs. “Family Check Up” (FCU) is a family intervention with a flexible approach to which topics are addressed, based on the family’s needs [46]. A study of the program showed that although early behaviour related to CU (at the age of 3) predicted negative development, the treatment outcome from the program was no lower for those with a high CU [47]. This indicates that even though children with CU traits score higher for behavioural problems both before and after treatment, participation in this program tailored to the family’s specific needs provided same level of reduction in problematic behaviour as for the children without CU traits.

This shows that in general, well-founded parent training interventions can contribute to reducing behavioural problems, also in children with CU traits. It does appear, however, that some elements of these programs do not have the expected effect, and more individualised and intensified treatments are needed to address the specific limitations related to CU traits. As previously stated, children and adolescents with CU traits are less sensitive to negative sanctions from others and to negative consequences of their actions. This means that, to an even greater extent, parent training measures for these children should emphasise warmth, cooperation, and encouragement based on the child’s positive interests, as a way to help the child reduce their problematic behaviour. Research has also shown that a structured empathy exercise that the child performs together with the parents improves the treatment outcome for children with CU traits [8]. The inclusion of such exercises could thus be a relevant element in the treatment of children with CU traits.

Another example of treatment advancements for younger children with CU traits is a tailored CU specific version of Parent Child Interaction Therapy (PCIT-CU). This program specifically emphasizes reward-based strategies over punishments, coaches’ parents to respond in a warm emotional manner, and aims to support the child’s development of emotional skills. A pilot study showed significant reductions in behaviour problems and increases in empathy among 23 children with conduct problems and elevated CU traits [48]. The fact that reductions in CU traits were observed in an effectiveness trial of another adaptation of PCIT directed at internalizing problems, suggests that the treatment components related to moral behaviour and emotion understanding might be sufficient to reduce CU traits [49]. Further research on the effect of various treatment components of parenting programs will advance the level of specificity of interventions appropriate for children with CU traits.

The treatment malleability of CU traits in children has also been observed in other studies. Research by Hawes and Dadds [44,45] showed a reduction in CU traits from before to after participation in the parent training. This change was maintained in follow-up six months later. In a randomised controlled study (RCT) in which parent training intervention in the home was compared with ordinary services (the sample was 66 families with children aged between four and nine), the experimental group had a reduction of psychopathy scores from before the start of treatment to after treatment, with a large effect size (d = 0.95). The reduction was maintained after 20 months, with an equivalently large outcome size (d = 0.89). More in-depth analyses showed that reductions in maternal harsh and inconsistent discipline were linked to a reduction in CU traits [50].

Similar reductions in CU traits were seen in a randomised control study (RCT) of an intensive parent training program for children between the ages of three and five years. The program focused on both parents’ and children’s self-regulation skills, and showed reductions in CU score of d = 0.85 from before until after treatment, with these changes being maintained one year later [51]. This demonstrates that sustainable reductions in CU traits are achievable through specific and tailored psychosocial interventions.

An attempt to identify specific treatment components related to reductions in CU traits for school-aged children was made in a study compiling pooled data from three randomized controlled effectiveness trials evaluating different treatment options within a municipality-based prevention program [52]. One treatment program was a shorter group-based course on parenting, namely Brief Parent Training. The second treatment option was the more extensive PMTO program. The third treatment program was individual social skills training (ISST), where school or kindergarten staff work individually with children identified to have behavioural problems. The participants were 550 families that included children (aged 3–12 years) with exhibited conduct problems (e.g., aggression or delinquency) at home, kindergarten, or school. Measurements were made both before and after treatment, as well as through a follow-up investigation six months after the completion of treatment (for more details on the RCT’s, see [53,54,55]).

The results from this study showed a positive change in CU traits across treatment for both parent training programs, but not for the social skills training. This points to the central role played by parents in creating change for children with CU traits. Moreover, it seems that individual social skills training given alone is probably not sufficient to stop the development of CU traits. With respect to sustained effects, only the comprehensive parent training model (PMTO) had a significant and direct impact on the children’s CU traits at the six-month follow-up. This indicates the need for extensive specialised parent training to achieve a permanent reduction of CU traits. Supplementary analyses showed that improved positive parenting skills (such as positive involvement, warmth, and praise/reward) were the change mechanism that led to a change in CU traits, while the reduction of negative parenting skills (such as harsh discipline) did not, similar to the results with the aforementioned studies. Improved parenting skills that are practised through PMTO can thus lead to a change in the child’s CU traits in a more positive direction. The PMTO program puts fundamental emphasis on positive involvement, praise, and encouragement as the most important parenting competence, which is in line with the knowledge of these children’s needs. A change in negative parenting impacts the level of behavioural problems in children without co-occurring CU, but showed no significant correlation with changes in CU traits in this study.

Overall, these studies give grounds for optimism, as they show that CU traits in children can be influenced through parent-oriented treatment programs. The relationship between parental warmth, positive involvement, and CU traits may in part explain this. For most parents, it seems natural and appropriate to be able to set protective limits for their child and to impose fair sanctions/consequences for repeated negative behaviour. If a child has clear CU traits, however, the research indicates that these parenting strategies might contribute little to the child’s positive development and acquisition of social interaction skills. In such situations, parents may need extra support to be reminded that their child might not benefit from boundaries and consequences in the same way as other children, and that attempting to maintain this can make it difficult to maintain the positive involvement that could provide better opportunities to teach their child more positive forms of interaction.

## 4. Treatment of Adolescents with CU

As previously noted, there are several well-established, research-supported treatment programs for adolescents with behavioural problems [7]. In general, these programs show that serious behavioural difficulties in adolescents can be treated through intensive, family-focused, and individually adapted treatment pathways. Several programs originate from the USA and have been implemented in a number of European countries. Multisystemic Therapy (MST) and Functional Family Therapy (FFT) are two of the three most widely used Blueprint model programs [56]. Even though research provides good support for the outcome of these treatment programs in general, until recently, few studies have investigated whether their effectiveness is affected by concurrent CU traits. We will present findings that shed light on this topic.

### 4.1. Multisystemic Therapy and CU Traits

Multisystemic Therapy (MST) is a family- and network-focused treatment model for adolescents with behavioural difficulties [57]. The model has been developed from research into the risk and protection factors for adolescent behaviour problems, which has shown that the most important dynamic (changeable) risk factors relate to parenting practices; interaction in the family; and contact with peers, school, leisure activities, and the immediate surroundings. The MST model provides a systematic, research-based framework for individually adapted treatment planning, based on rigorous multi-systemic analyses and the continuous prioritisation and evaluation of therapeutic interventions. At the start of MST, there is active and intense work on forming a good cooperative alliance with the parents and adolescent, as well as a detailed multi-informant assessment of current risk and protective factors. The assessment involves all relevant systems, including the adolescent and the parents, as well as the adolescent’s teacher and other important key people in the surroundings (e.g., a neighbour or aunt). Based on analyses and prioritisation of risks and protective factors in the adolescent’s ecological contexts (such as many negative friends, drug use, and absence from school), therapeutic interventions are developed with the aim of changing these conditions for the better. MST is an intensive treatment program with up to several meetings a week, and with a duration of 3–5 months.

Several international studies support the effectiveness of MST for adolescents with severe behavioural difficulties [58,59,60]. To date, two studies have specifically considered the effects of MST on adolescents with CU traits [61,62]. The studies generally show that adolescents with a high degree of CU traits also benefit from MST treatment. The Dutch study shows that the control group also achieves a reduction in problematic behaviour [62]. Yet, the article provides little information about the treatment and measures received by the control group. It is therefore difficult to interpret whether this means that the control group received another treatment that was also effective for youth with CU traits, or whether both groups showed a normal decline from a high level to a more moderate level of behavioural problems as part of natural variation over time. The studies are also not clear on the malleability of adolescent CU traits as a consequence of MST treatment. One of the studies observed reductions in parent-reported CU, but not in self-reported CU [61].

### 4.2. Functional Family Therapy and CU Traits

Functional Family Therapy (FFT) is a family therapeutic treatment model for adolescents with behavior problems. As with MST, the model builds on the knowledge base of risk and protection factors for adolescent behaviour problems. In FFT, the therapist addresses poor communication and dysfunctional interactions in the family. The treatment focuses particularly on creating motivation and hope in the first part of treatment. The therapist actively reaches out to get everyone to attend sessions, and meets the family with a respectful, non-judgmental and strength-based, family-focused attitude and behaviour, so as to reduce guilt and blame within the family and increase their sense of hope. When a more positive family atmosphere and motivation for change has been created, the therapist helps the family to practice more constructive and supportive communication, and teach them new interaction skills such as problem solving, negotiation, and conflict management. FFT places an emphasis on family members being able to use new skills in a manner that does not change the way each of them experiences a level of contact or autonomy they feel comfortable with [63].

FFT has also been the subject of several international studies that show positive treatment results [59,64,65]. Only a few of these studies have specifically considered the significance of CU traits. There are grounds to believe, however, that a treatment model such as FFT can be particularly useful for adolescents with CU [66]:

- FFT takes a strength-focused and relational approach to the entire family. Together, the adolescent and the parents will participate in the change process, and are motivated by a respectful and non-judgmental attitude on the part of the therapist.
- FFT works actively to teach the family skills that lead to good and positive family relationships. The focus is on strengthening the family’s cohesion and their respect for each other. The family practises using positive and constructive, rather than negative and critical, forms of communication.
- FFT focuses on increasing the parents’ ability to see and emotionally understand the adolescent in a caring way.
- FFT has a strong focus on ensuring that young people and parents can cooperate positively through respectful dialogue, mutual agreements, praise, and encouragement.
- FFT attaches great importance to matching the interventions to the needs of each individual family and adolescent.

In an American study of FFT, researchers found a reduction in both behavioural problems and the number of new arrests for adolescents with high CU scores at follow-up 6 and 12 months after the conclusion of treatment [66]. As for the MST study, this showed that adolescents with CU traits can benefit from the treatment program. Despite this reduction, however, adolescents with CU traits still showed a significantly higher level of problematic behaviour after treatment than the adolescents without CU traits. The CU score was also related to the number of new offences during treatment. There was no correlation, however, between CU traits and participation in and the completion of treatment. The FFT model has a strong focus on ensuring the families’ participation and motivation, and it seems that the method also does this successfully for young people with CU traits.

The results of a Danish pre-post treatment study showed that even though CU traits were associated with more rule-breaking behaviour and hyperactivity at the start of treatment, this did not increase the risk of adolescents interrupting or gaining less benefit from FFT [67]. On the other hand, CU traits were related to the level of improvement as rated by parents, adolescents, and the therapist at the end of treatment. Here, pre-treatment CU traits predicted slightly reduced ratings of the improvements made with respect to parental supervision. Equivalent to the study by White et al. [66], the results indicated that although individuals with CU traits undergo by and large the same change as other adolescents, they still have a higher level of problems at the end of treatment.

This research does provide support for established treatment models for adolescents with behavioural difficulties to have a general treatment effect on behavioural problems for adolescents displaying CU traits. Interestingly, the therapeutic alliance, which is important in psychotherapy in general, might seem to have an even greater impact on treatment outcomes for adolescents with CU traits compared to adolescents without these traits [68]. This may explain the positive results from MST and FFT for this target group, as both models work actively to ensure a therapeutic alliance with both parents and adolescents.

## 5. Conclusions

Children and adolescents who show signs of violating the general social expectations of acting empathetically and showing consideration for others are a source of concern. The ability to regulate one’s own behaviour in relation to the needs and feelings of others is such a fundamental aspect of human interaction that any violation of this leads to unrest, irritation, and sometimes aggression. As an element of normal upbringing, it is natural to set boundaries for this type of behaviour and to make the child aware that behaviours that are detrimental to other people will have negative consequences. Most children will learn from this feedback, develop empathy, and increasingly use this to regulate their behavior.

Some children and adolescents repeatedly show a lack of this ability, which, in a severe and persistent form, is referred to as CU traits. It is interesting that research has shown that the most natural reactions to these children’s behaviour (e.g. reprimands, boundary setting, and negative consequences) appear to have the least effect on their behaviour. On the contrary, research indicate that the path to positive regulation of these children’s behaviour lies in positive and warm relationships. They seem less affected by negative consequences (e.g., time-out), but respond well to positive encouragement (e.g., rewards). As these children are primarily motivated by self-interest, the best way to influence them appears to be making sure that they like the people around them and therefore choose to show (self-) consideration for them as someone they like.

Given that what these children might need is quite different from the reactions they typically receive, it is important to have specialised and professionally updated treatment programs for this group. On a general basis, it is important to emphasise that several of the existing research-based treatment programs for children and adolescents with behavioural difficulties seem to have a positive effect on this group. In particular, a prevention perspective can be important, as those who receive treatment early appear to achieve a noticeable and sustainable reduction in CU. There are promising results from applying strategies to reward good behaviour—notably by giving the reward immediately. In parent training aimed at children with CU traits, it seems to be important to support and motivate parents to maintain a warm relationship with a focus on positive encouragement. Special adaptations of parenting programs are also showing encouraging results. When it comes to slightly older children and adolescents (from 11 to 17 years old), it seems that both FFT and MST can work relatively well for this group. Both methods are based on in-depth analyses and individual adaptation of the treatment, as well as a focus on creating positive family relationships and a developmentally supportive environment. In the FFT model, there is also focus on ensuring effective collaboration strategies in which the adolescent can be included as an equal party, and this may be of importance to this target group. Interventions developed specifically to target CU trait reduction are also of great interest in order to diminish the risk that CU traits can develop into adult psychopathy and life-course persistent anti-social behaviour.

Even though already established programs may claim treatment effects for young people with CU traits, there may be a need for the further development of these programs. It may also be important to gain more knowledge of how to forge a good alliance with adolescents with CU traits, so that the treatment can take greater account of the unique challenges faced by adolescents with CU traits. There is ongoing research aiming to identify which treatment elements seem to be particularly effective for children and adolescents with CU traits. This research might contribute to greater customization of existing treatment programs, and to developing completely new treatment programs for the CU group. When directed at children with CU traits, parenting programs should emphasize boundary setting as a last-resort strategy and should focus even more on affection, praise, and reward as a means to influence the child’s or adolescent’s behaviour [69]. Possible additions to existing programs might be different forms of empathy training, either via the parents or directly with the child. In any attempts to train children and adolescents with CU traits, such as in empathy and the ability to form a perspective, it is probably most effective to focus on an understanding of empathy, rather than the feeling of empathy. At noted previously, such developments have been made with the PCIT-CU program for children. Socializing children and adolescents with CU traits to (1) understand empathy for others, (2) be able to choose to act empathetically based on this understanding, and (3) themselves experience positive and beneficial effects, can be a means to promote prosocial behaviour. This form of empathy training has been proven to have good treatment effects for children with CU traits [8]. In this study, the researchers reflected that it might have been significant that the exercises took place through interactions between the child and the parents. The study that showed the importance of a therapeutic alliance for adolescents with CU traits also pointed to how it is the positive relational aspects of human interaction that seem to have a positive effect on children and adolescents with CU traits.

A promising development made for the treatment for adolescents with CU traits is the PSYCHOPATHY.COMP program, based on motivational interviewing strategies and Compassionate Focused Therapy (CFT) [70,71]. The program focuses on helping youth to reconcile with the notion that although much is determined by a range of external influences we have no control over (e.g., evolutionary, genetic, epigenetic, and environmental factors), everybody has a responsibility to act in prosocial ways. During sessions, the adolescent is introduced to CFT practices that diminish threat responses, increase emotion regulation, and instil soothing and compassionate feelings and actions. A pilot study of the program has shown promising results in reducing psychopathic traits [71]. Furthermore, it seems that the inclusion of the youth in treatment, although challenging, might be a key element in providing effective care [72]. To our knowledge, the studies of the treatment of children and adolescents with CU traits have not looked at potential effects stemming from parental psychopathy. Given that CU traits are highly heritable and, to some extent, could be influenced by genetics, one should assume that parents of children with CU traits would have some degree of CU traits themselves. It is likely that parental CU traits might lower their motivation for and compliance with parent training and family therapy aimed to help their child, but we lack empirical data on this. Moreover, few studies of interventions for preventing the development of psychopathic traits among children and adolescents have follow-up data beyond a year. Future research is therefore needed to examine whether treatment gains persists into adulthood.

## Data Availability

Not applicable.
